# Supplementary figures and images for: The p97/VCP segregase is essential for arsenic-induced degradation of PML and PML-RARA
Source: J Cell Biol. 2023 Feb 28;222(4):e202201027. doi: 10.1083/jcb.202201027 (PMC10005898; doi:10.1083/jcb.202201027)

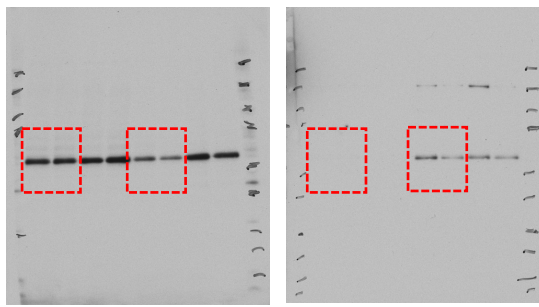

Fig. 1D (p62)

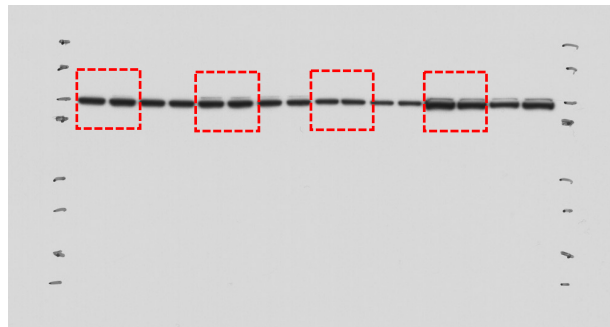

Fig. 1D (p97)

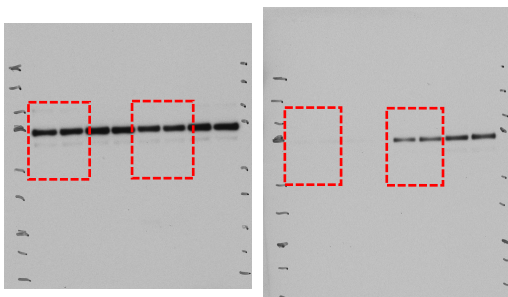

Fig. 1D (PIAS1)

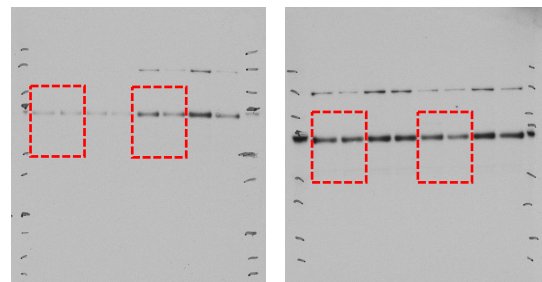

Fig. 1D (SENP1)

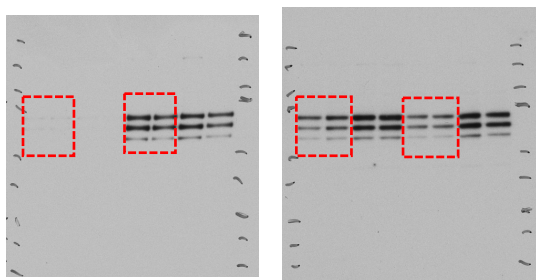

Fig. 1D (PIAS3)

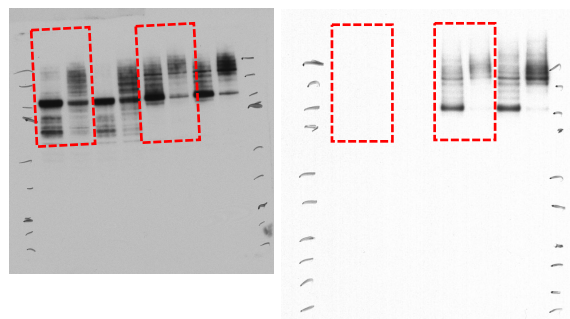

Fig. 1D (PML)

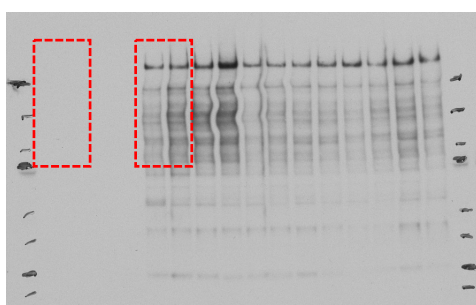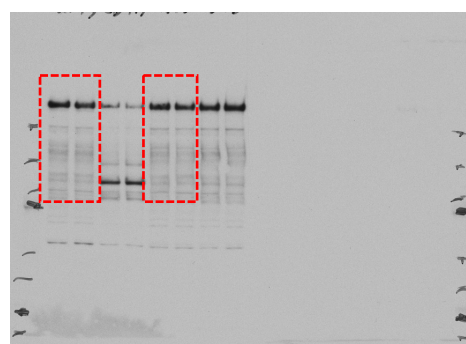

Fig. 1D (SETX)

Supplement: SourceData F1 — is the source file for Fig. 1. [file JCB_202201027_SourceDataF1.pdf]

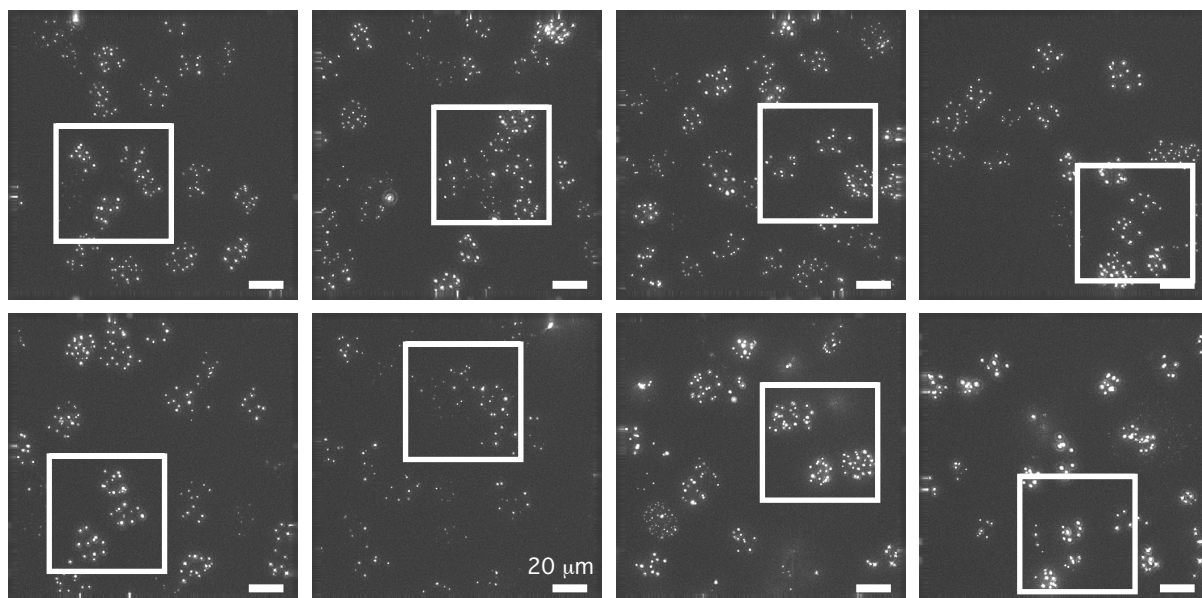

Supplement: SourceData F3 — is the source file for Fig. 3. [file JCB_202201027_SourceDataF3.pdf]

Fig. 4C

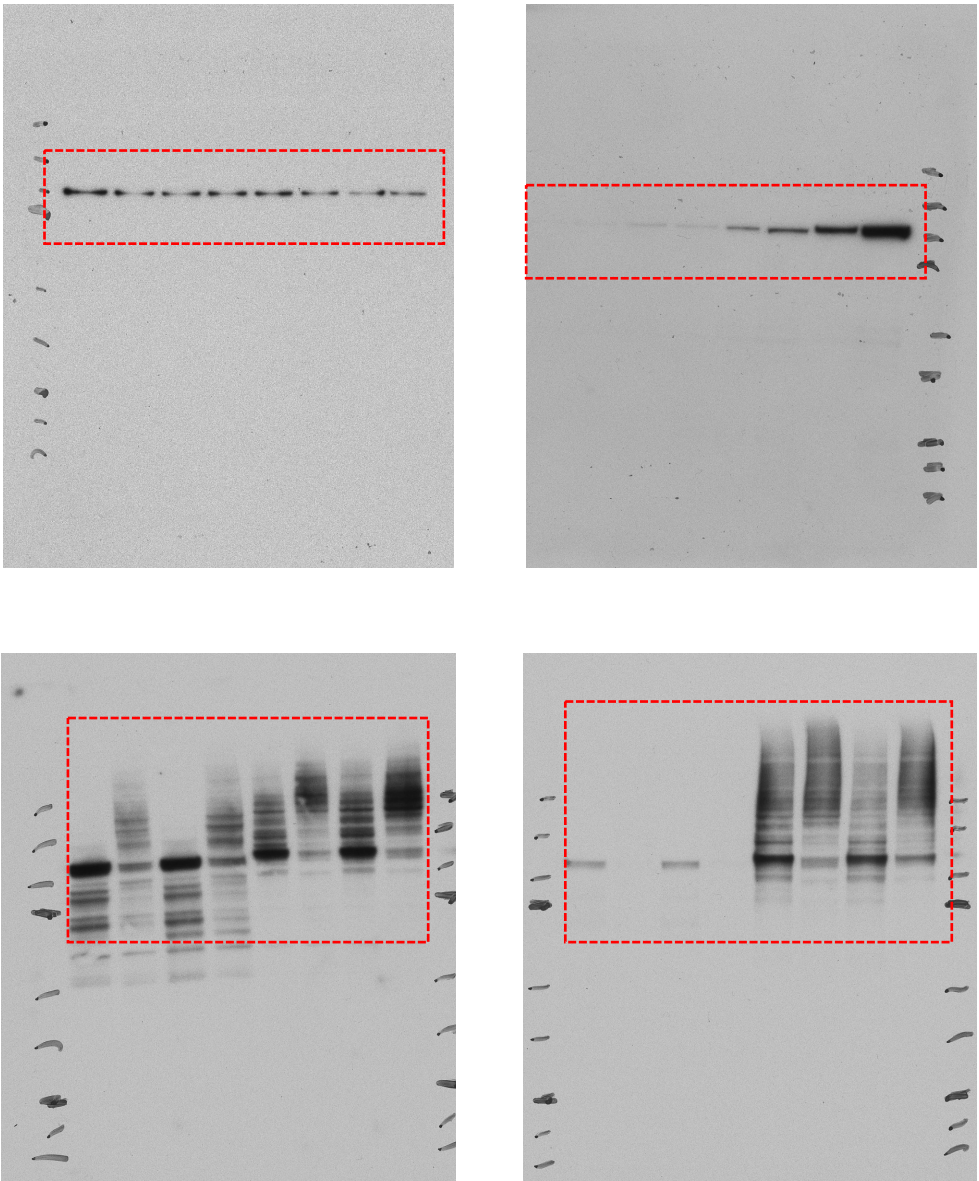

Supplement: SourceData F4 — is the source file for Fig. 4. [file JCB_202201027_SourceDataF4.pdf]

Fig. 5 A

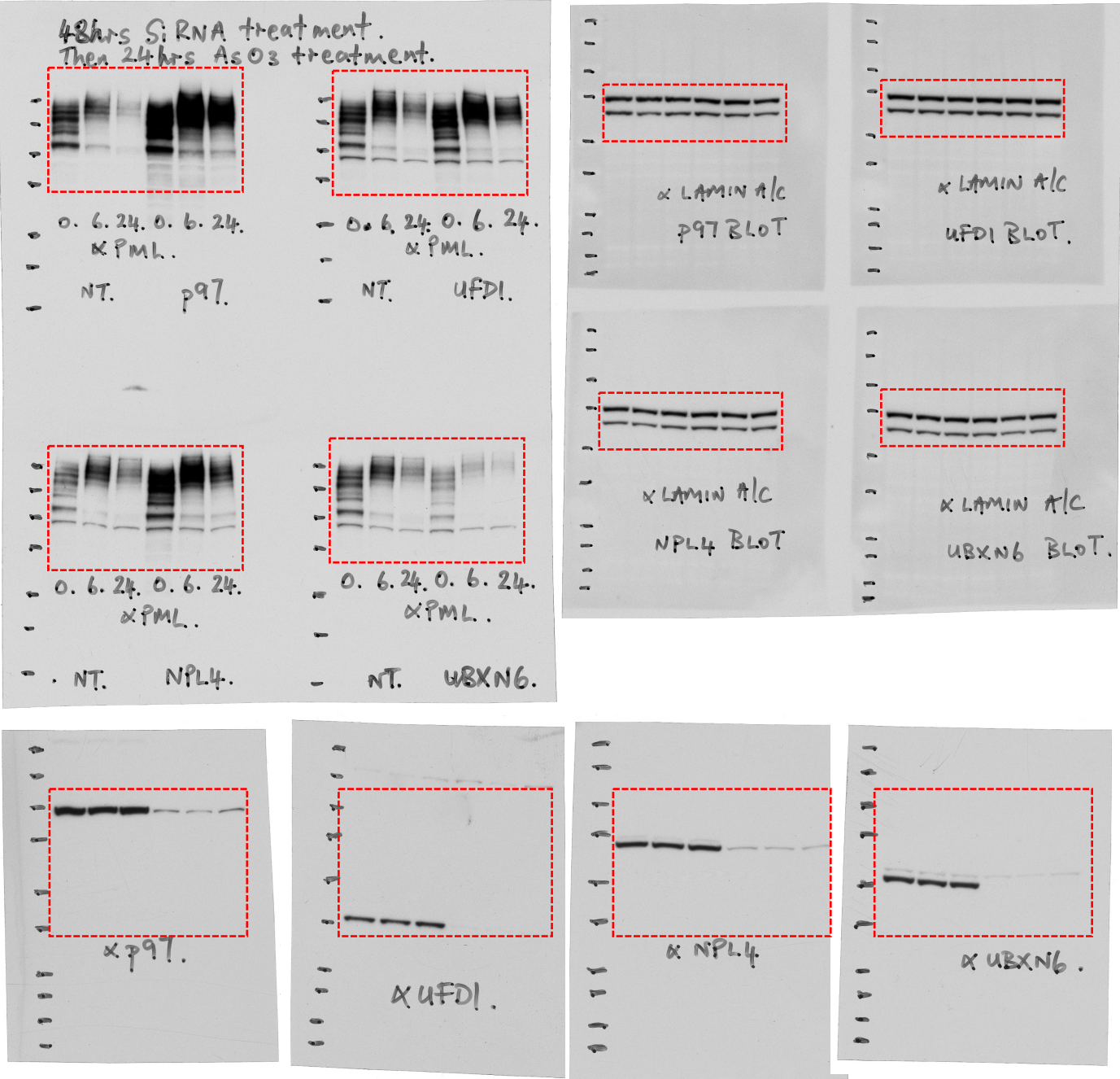

Fig. 5B

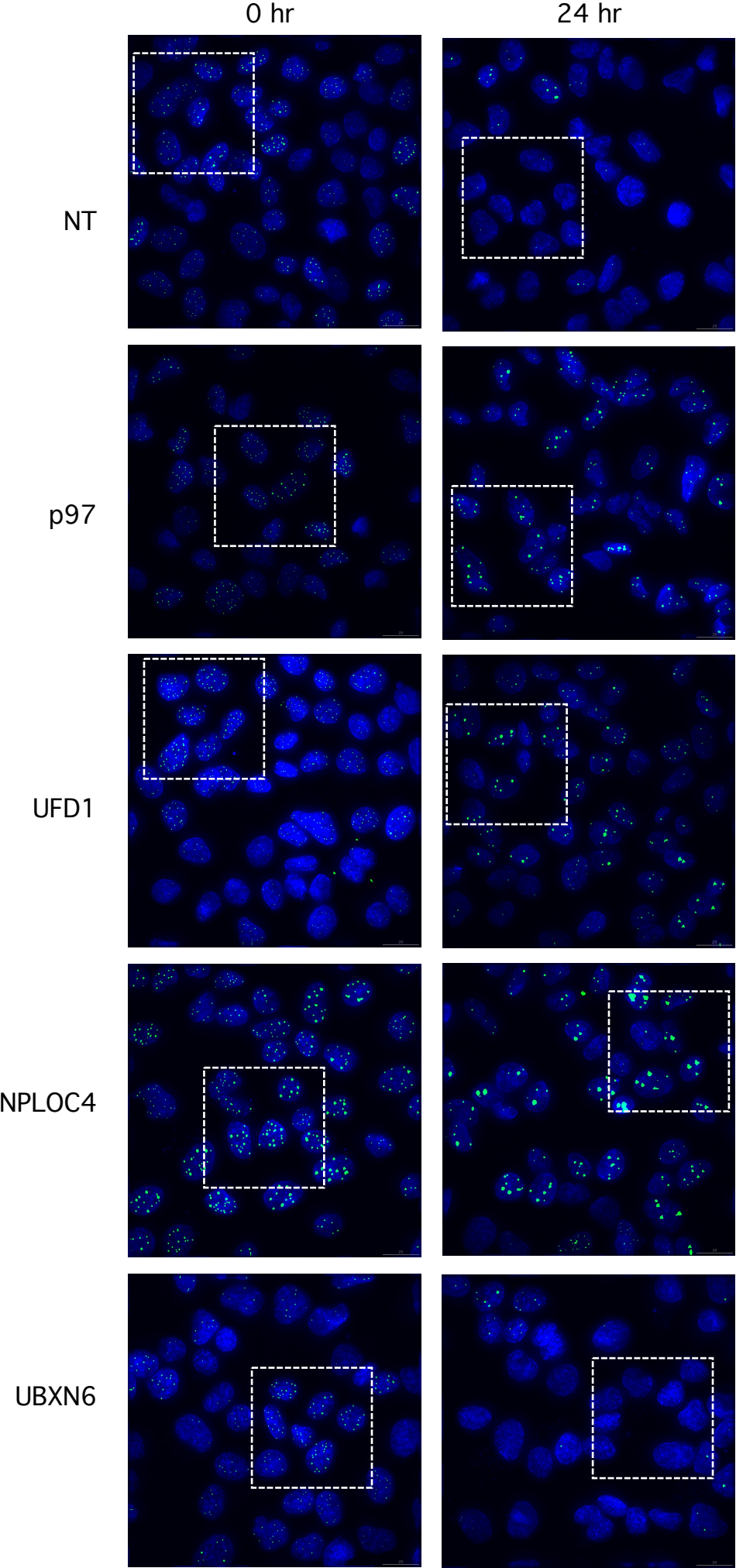

Supplement: SourceData F5 — is the source file for Fig. 5. [file JCB_202201027_SourceDataF5.pdf]

Fig. 6 A

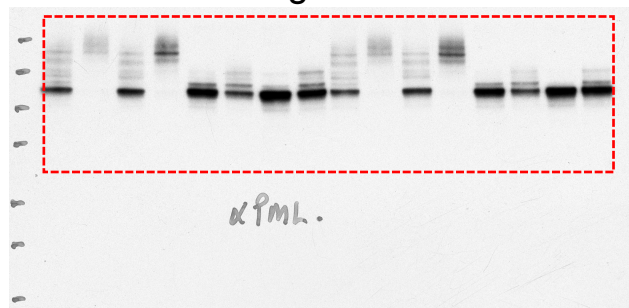

Fig. 6 D

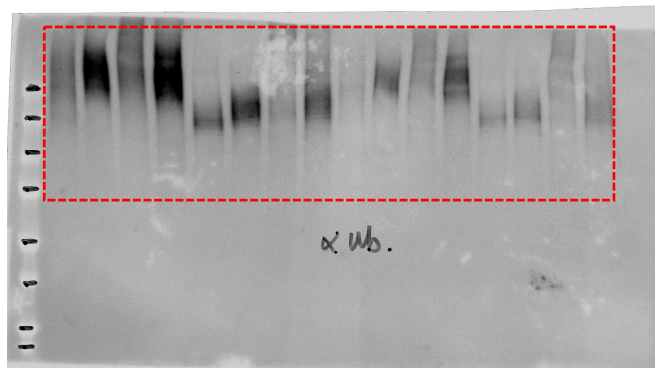

Fig. 6 B

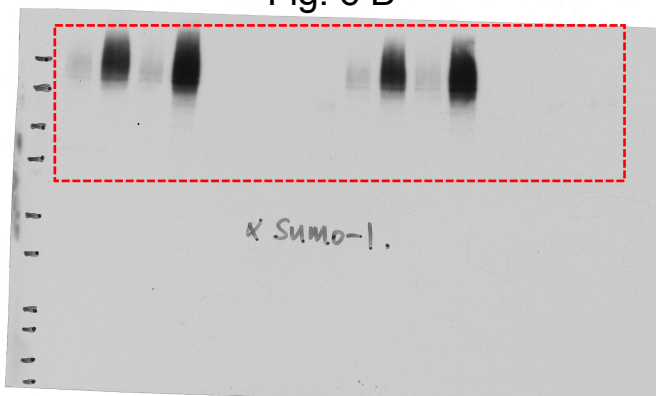

Fig. 6 E

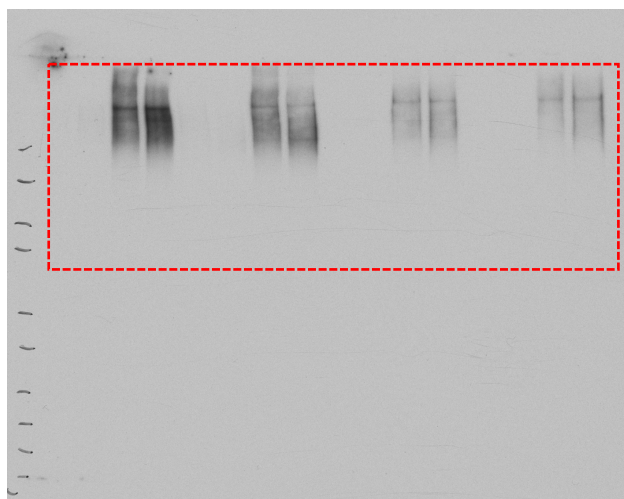

Fig. 6 C

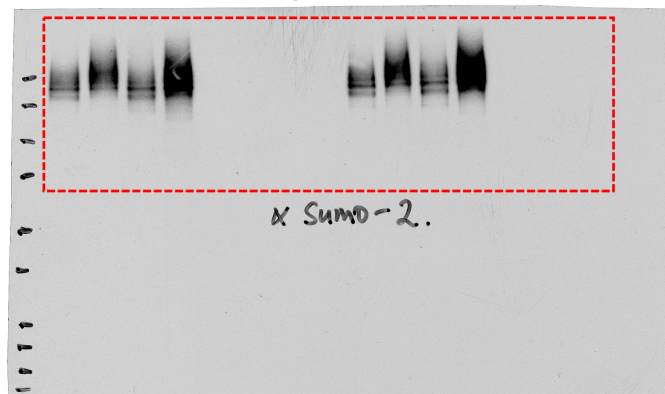

Fig. 6 F

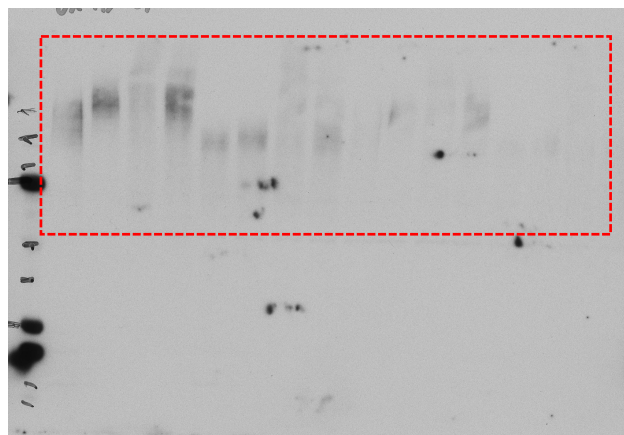

Supplement: SourceData F6 — is the source file for Fig. 6. [file JCB_202201027_SourceDataF6.pdf]

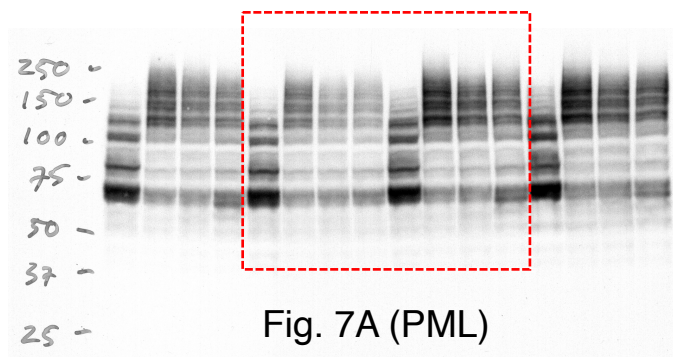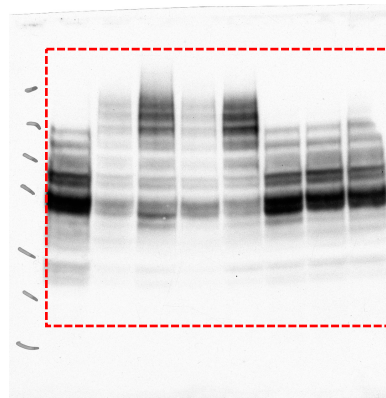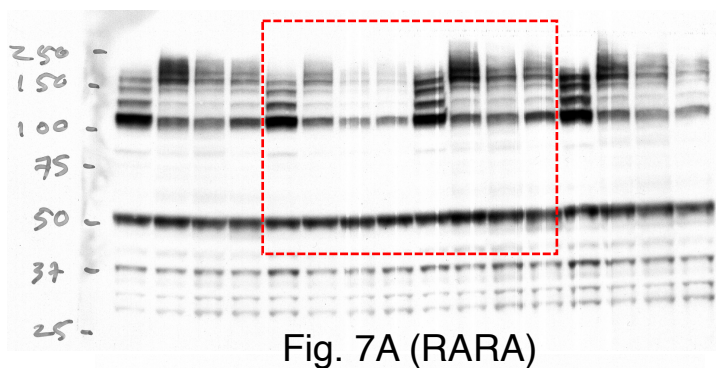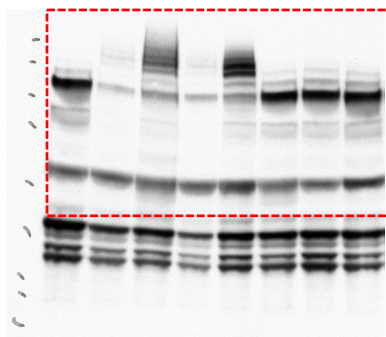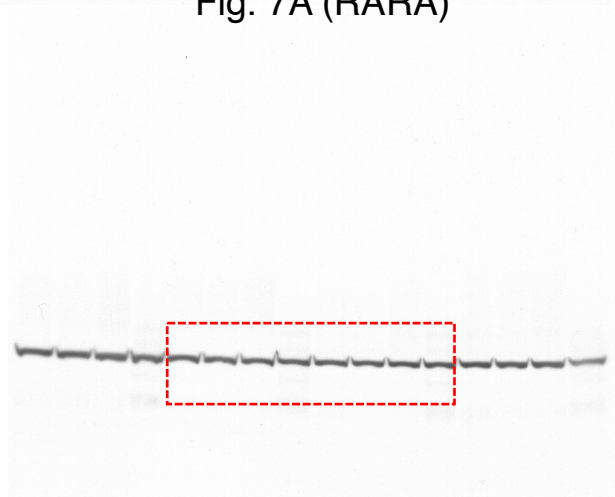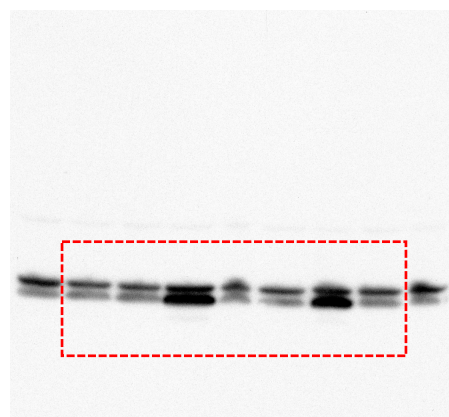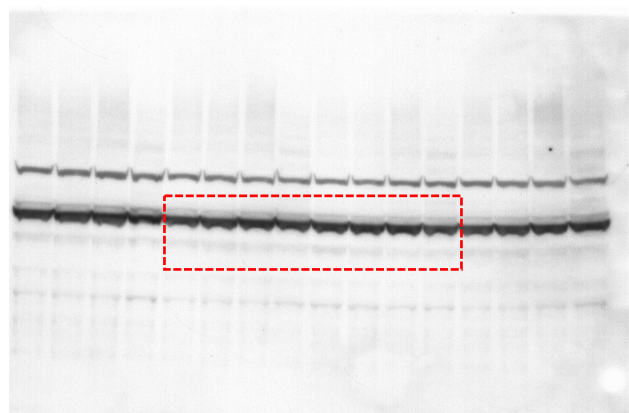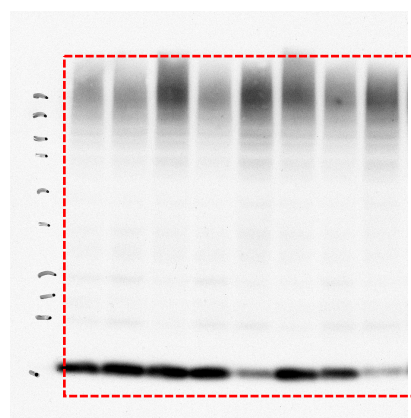

Supplement: SourceData F7 — is the source file for Fig. 7. [file JCB_202201027_SourceDataF7.pdf]

Supp. Fig. 1A

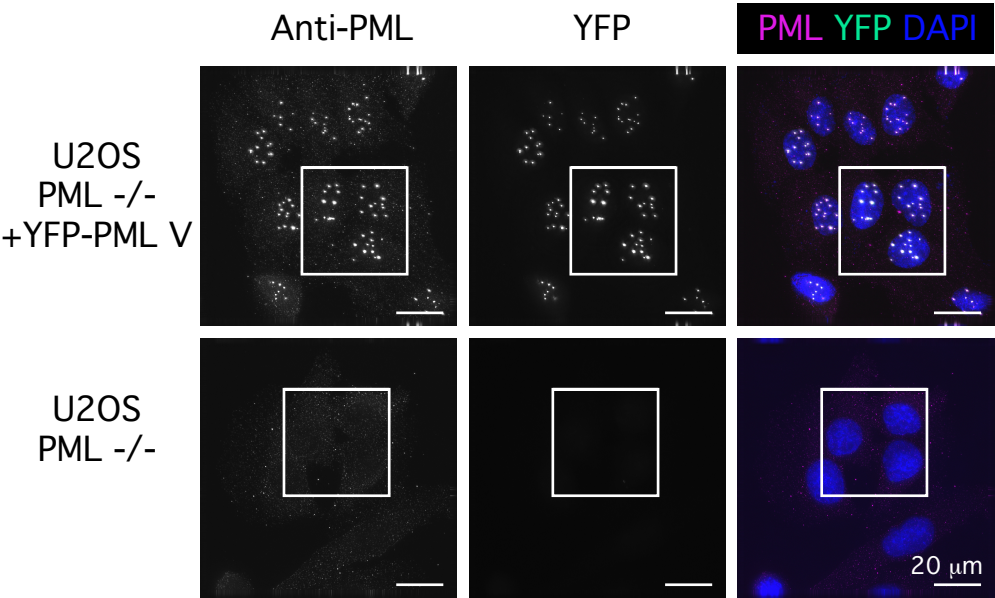

Supp. Fig. 1B (Left)    Supp. Fig. 1B (Right)

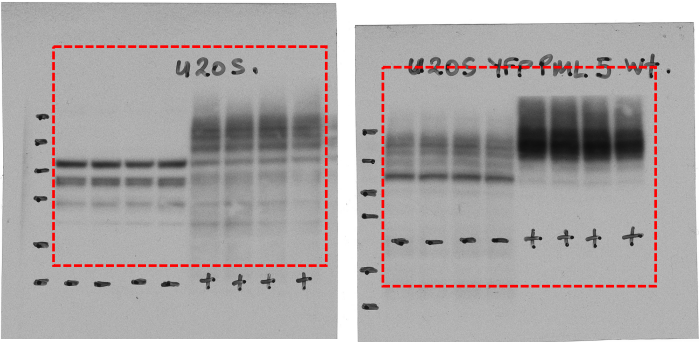

Supp. Fig. 1D (Lower)

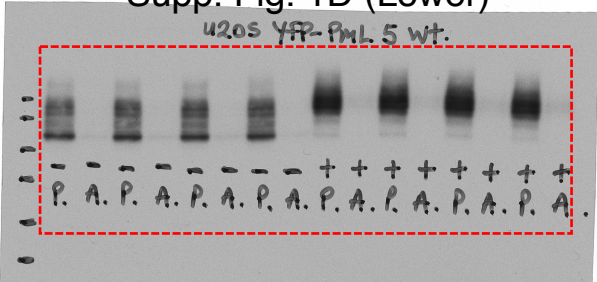

Supp. Fig. 1E

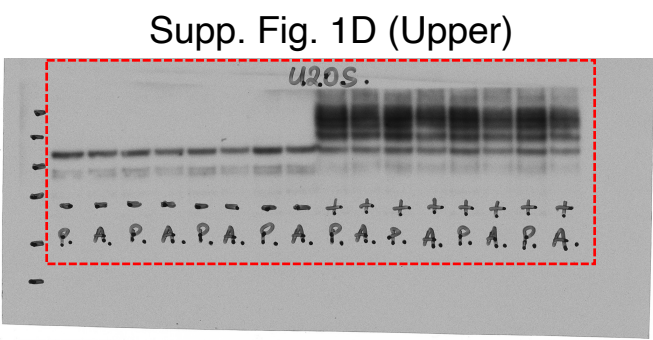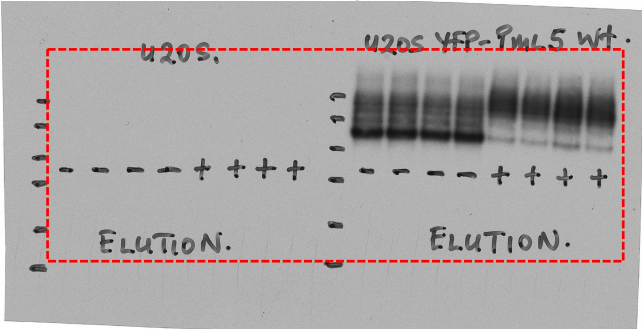

Supplement: SourceData FS1 — is the source file for Fig. S1. [file JCB_202201027_SourceDataFS1.pdf]
