# Supplementary material for: The p97/VCP segregase is essential for arsenic-induced degradation of PML and PML-RARA
Source: J Cell Biol. 2023 Feb 28;222(4):e202201027. doi: 10.1083/jcb.202201027 (PMC10005898; doi:10.1083/jcb.202201027)

Fig. 2A (upper)

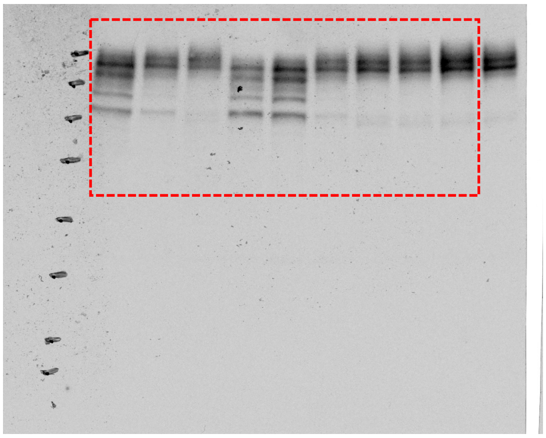

Fig. 2B (upper)

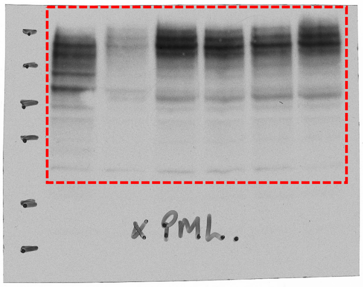

Fig. 2A (lower)

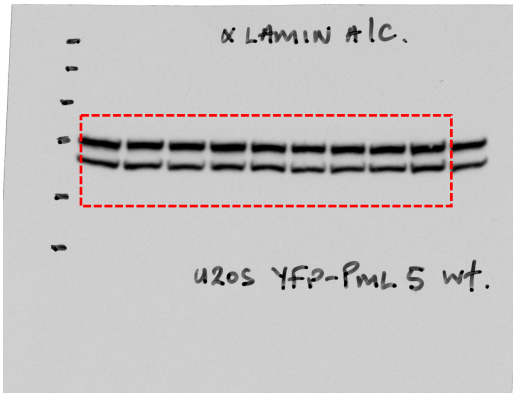

Fig. 2B (lower)

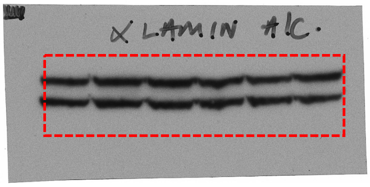

Fig. 2C (upper)

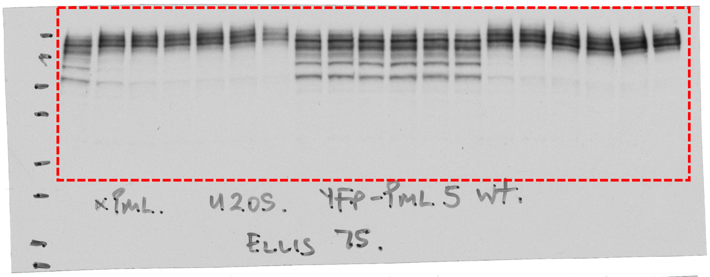

Fig. 2 C, D & E (Lamin A/C blots)

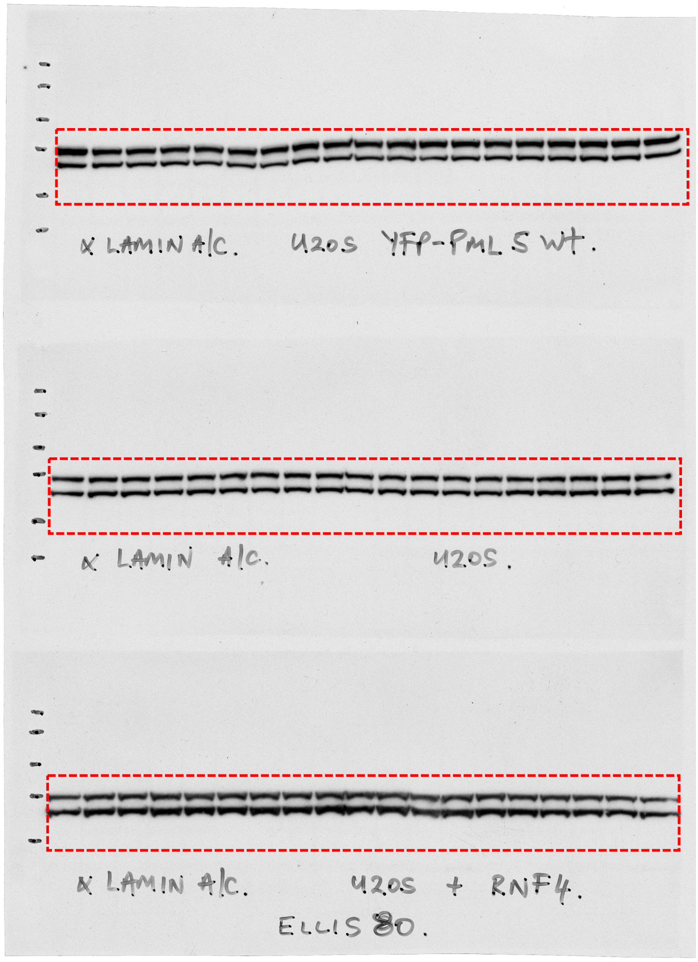

Fig. 2D (upper)

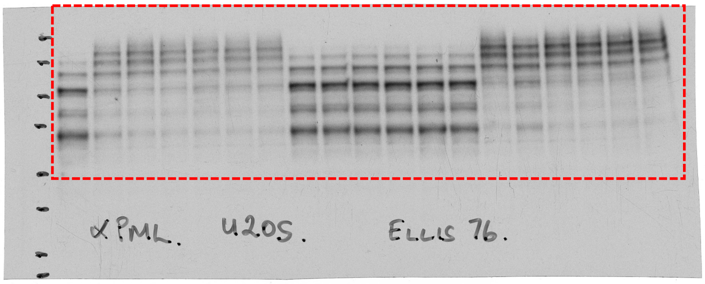

Fig. 2 E (upper)

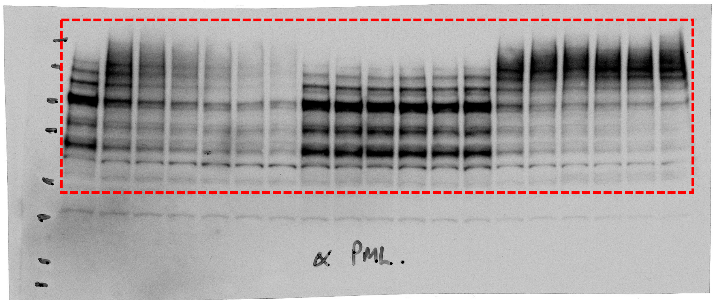

Supplement: SourceData F2 — is the source file for Fig. 2. [file JCB_202201027_SourceDataF2.pdf]
