# Supplementary material for: The p97/VCP segregase is essential for arsenic-induced degradation of PML and PML-RARA
Source: J Cell Biol. 2023 Feb 28;222(4):e202201027. doi: 10.1083/jcb.202201027 (PMC10005898; doi:10.1083/jcb.202201027)
Supplement: SourceData FS2 — is the source file for Fig. S2. [file JCB_202201027_SourceDataFS2.pdf]

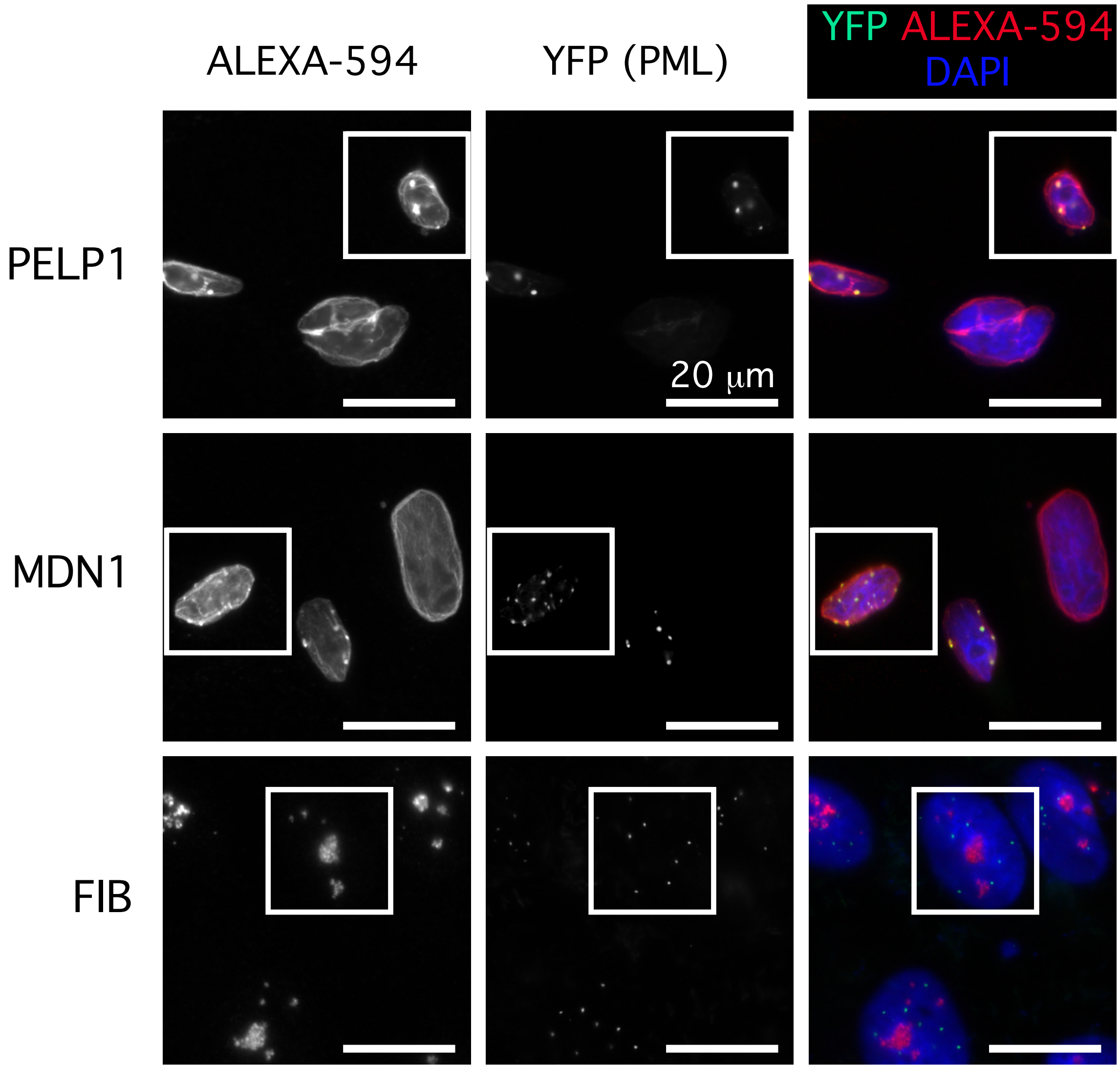
ALEXA-594
YFP (PML)
YFP ALEXA-594
DAPI
PELP1
MDN1
FIB
20 μm

Figure_2023-2-10_13:23:13 Sup2 G

Link to Figure: https://nightshade.openmicroscopy.org/figure/file/387160/

Figure contains the following images:

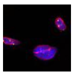

58_YFPPML_ATO_PELP1_02_R3D.dv
https://nightshade.openmicroscopy.org/webclient/?show=image-4550216

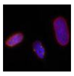

59_YFPPML_ATO_MDN1_05_R3D.dv
https://nightshade.openmicroscopy.org/webclient/?show=image-4550207

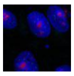

U2OS_YFPPML_ATO_FIB_01_R3D.dv
https://nightshade.openmicroscopy.org/webclient/?show=image-4550217

Scalebars:

Scalebar Lengths: 20 µm
