# Supplementary material for: The p97/VCP segregase is essential for arsenic-induced degradation of PML and PML-RARA
Source: J Cell Biol. 2023 Feb 28;222(4):e202201027. doi: 10.1083/jcb.202201027 (PMC10005898; doi:10.1083/jcb.202201027)

Supp. Fig. 3A (upper)

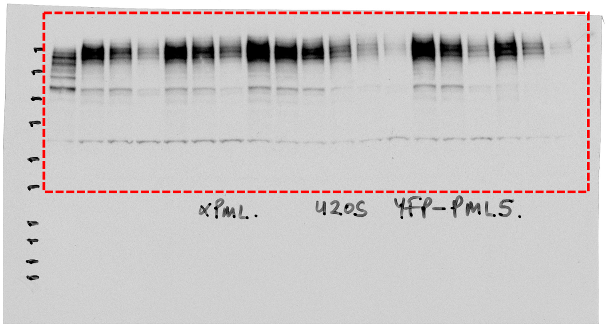

Supp. Fig. 3A (lower)

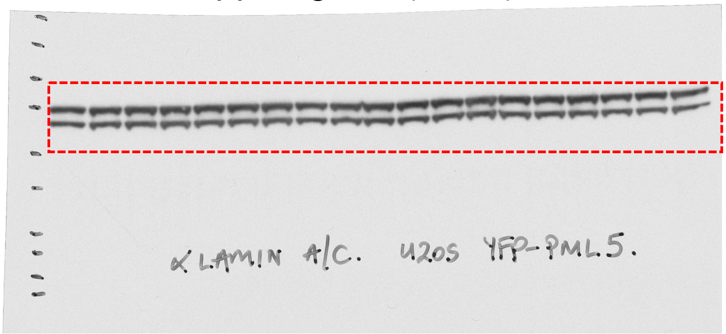

Supp. Fig. 3B (UFD1)

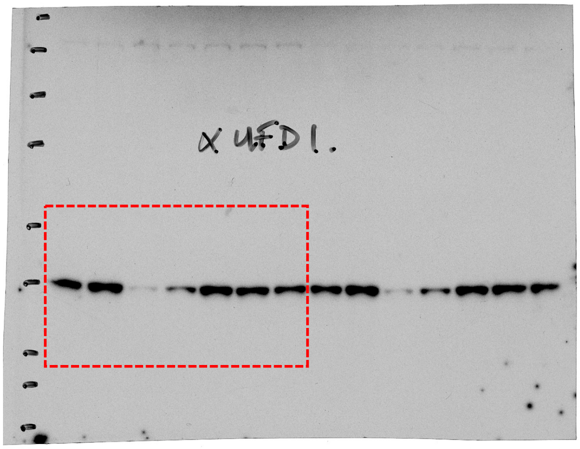

Supp. Fig. 3B (NPLOC4)

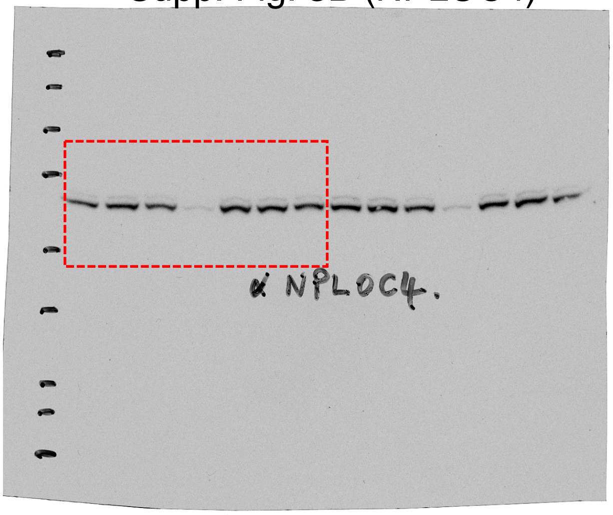

Supp. Fig. 3B (UBXN6)

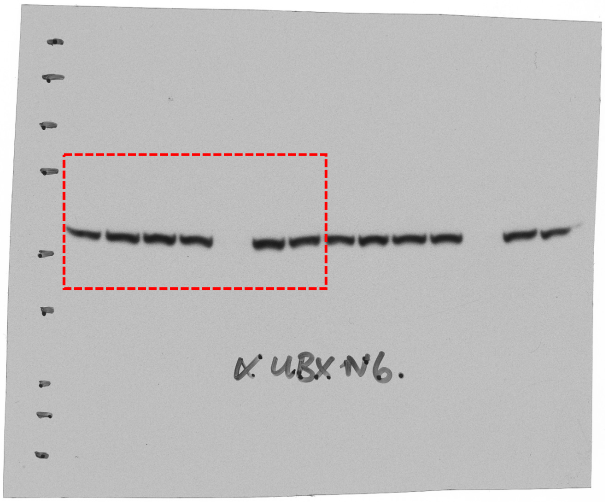

Supp. Fig. 3B (FAF2)

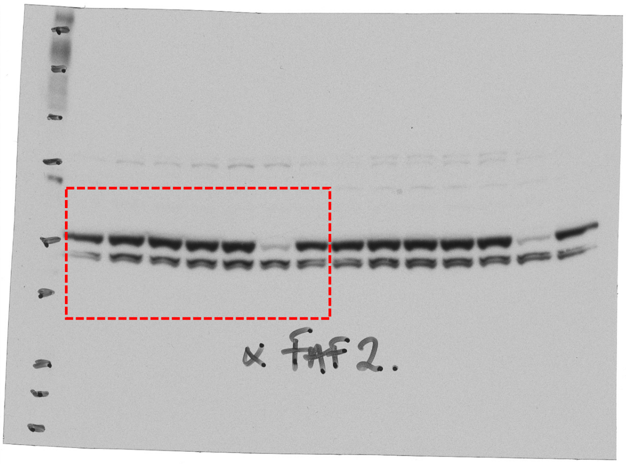

Supp. Fig. 3B (PLAA)

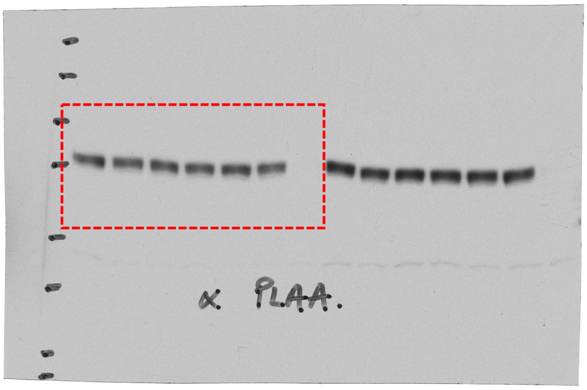

Supplement: SourceData FS3 — is the source file for Fig. S3. [file JCB_202201027_SourceDataFS3.pdf]
